# Diagnostic yield, safety, and molecular-era clinical utility of stereotactic biopsy for brainstem lesions: a contemporary systematic review and meta-analysis

**DOI:** 10.3389/fsurg.2026.1953479

**Published:** 2026-09-09

**Authors:** Hanqiang Du, Jiajia Wang, Qicheng Liu, Gaoquan Luo

**Affiliations:** Department of Neurosurgery, General Hospital of the Southern Theater Command, Guangzhou, China

**Keywords:** brainstem, complications, diagnostic yield, meta-analysis, molecular testing, permanent morbidity, stereotactic biopsy, systematic review

## Abstract

**Background/objectives:**

Stereotactic biopsy of brainstem lesions must balance diagnostic and molecular information against procedure-related neurological risk. We updated the evidence base after a reviewer-directed literature coverage and overlap audit.

**Methods:**

This PRISMA 2020 systematic review and meta-analysis was registered in PROSPERO (CRD420261432357). Searches covered English and Chinese bibliographic databases, trial registries, and Google Scholar, with forward and backward citation tracking during revision. Diagnostic yield and overall complications were analyzed with logit random-effects models; permanent neurological morbidity and procedure-related mortality were analyzed with binomial-normal generalized linear mixed models (GLMMs). Molecular clinical utility was synthesized narratively because its constructs and denominators were heterogeneous.

**Results:**

Seventy-six reports met review-level eligibility (71 full-text and 5 abstract/conference reports), and 55 independent studies contributed to at least one preferred quantitative synthesis. Diagnostic yield was 1,814/1,956, with a pooled estimate of 93.5% (95% CI, 91.5%–95.0%). Overall complications were 162/1,797, with a pooled estimate of 10.5% (95% CI, 8.5%–12.8%). Permanent neurological morbidity was reported in 17 compatible studies (14/859; GLMM 0.83%, 95% CI, 0.22%–3.03%). Procedure-related mortality was 11/1,765 (crude 0.62%, exact 95% CI, 0.31%–1.11%); the primary GLMM estimate was 0.30% (95% CI, 0.07%–1.24%), whereas continuity-corrected models produced higher estimates. Diagnostic yield remained 93.7% (95% CI, 91.3%–95.5%) after excluding studies with fewer than 20 participants, although observed yield was inversely associated with study size.

**Conclusions:**

In selected published cohorts, stereotactic biopsy provided high diagnostic yield. Overall complications were clinically meaningful, while observed procedure-related mortality was below 1% and permanent morbidity was uncommon but imprecisely estimated. Population, era, technique, outcome definition, and small-study analyses support cautious individualized interpretation rather than a universal biopsy recommendation.

**PROSPERO registration:**

CRD420261432357

## 1 Introduction

Brainstem lesions remain among the most consequential diagnostic problems in neuro-oncology and neurosurgery. The midbrain, pons, and medulla contain densely organized long tracts, cranial nerve nuclei, and autonomic pathways, so even a small procedure-related injury may have substantial clinical effects. The radiographic differential diagnosis is broad and includes diffuse and focal glioma, lymphoma, metastasis, inflammatory disease, infection, demyelination, vascular lesions, and other mimics. Management therefore requires a patient-specific balance between the anticipated value of tissue and the risk of reaching the selected target (1–3).

Magnetic resonance imaging is central to this decision, but imaging does not eliminate diagnostic uncertainty in every patient. Adult series have shown that lesions considered radiologically compatible with glioma can represent tumors of different grades or entirely different pathologies, with corresponding changes in treatment. Tissue diagnosis may be particularly relevant when imaging is atypical, when the clinical course is discordant with the presumed diagnosis, or when a specific histological diagnosis would redirect therapy (4–6). These observations do not imply that all brainstem lesions require biopsy; rather, they identify settings in which diagnostic uncertainty can itself carry clinical risk.

The role of tissue has also changed with contemporary central nervous system tumor classification. Integrated diagnoses increasingly combine histological and molecular features, and diffuse midline glioma, H3 K27-altered, is a prominent example in which molecular status has diagnostic and prognostic relevance. Adequate tissue may also permit methylation profiling, sequencing, identification of potentially actionable alterations, or eligibility assessment for molecularly stratified trials (7, 8). The value of biopsy in this setting is therefore broader than conventional histological confirmation, although molecular feasibility should not be equated with proven survival benefit.

Technical practice has evolved in parallel. Frame-based stereotaxy remains established, while frameless navigation, robotic assistance, intraoperative imaging, and tailored transfrontal or transcerebellar trajectories have expanded procedural options. Contemporary series describe clinically usable tissue with each approach, but performance remains dependent on lesion anatomy, platform availability, pathology workflow, and center experience (9–15). The available evidence does not establish a universal technical hierarchy for all brainstem targets.

Evidence synthesis is complicated by small retrospective cohorts, different age groups, overlapping institutional series, changes in technology across eras, and inconsistent definitions of diagnostic yield and morbidity. Previous meta-analyses established high diagnostic yield with relatively infrequent permanent morbidity, but the apparently smaller evidence set in the submitted version prompted a full coverage and overlap audit during revision (1–3). An updated analysis should distinguish broad overall complications, permanent neurological morbidity, sparse mortality, and molecular clinical utility rather than collapsing them into one benefit-risk estimate.

Adult and pediatric evidence should not be treated as fully interchangeable. Pediatric series are dominated by diffuse intrinsic pontine or diffuse midline glioma, for which the historical imaging-based pathway and contemporary rationale for molecular profiling are prominent. Adult brainstem lesions have a broader pathological differential, and biopsy may more often resolve uncertainty among glioma, lymphoma, metastasis, inflammation, and other entities (4–6, 16). A combined synthesis can evaluate procedural performance, but interpretation should retain population differences.

We therefore performed a contemporary systematic review and meta-analysis of stereotactic biopsy for brainstem lesions. The objectives were to estimate diagnostic yield, overall procedure-related complications, permanent neurological morbidity, and mortality using an overlap-controlled evidence set; examine population, era, technique, diagnostic-definition, and small-study effects; and synthesize molecular-era clinical utility without forcing incompatible endpoints into a single pooled rate.

## 2 Methods

### 2.1 Study design and reporting

This systematic review and meta-analysis was registered in PROSPERO (CRD420261432357) and followed PRISMA 2020. The review question concerned adult and pediatric patients with brainstem lesions undergoing stereotactic or image-guided biopsy, with diagnostic yield, overall procedure-related morbidity or complications, permanent neurological morbidity, procedure-related mortality, and molecular-era clinical utility treated as distinct outcomes. The completed PRISMA checklist and flow diagram accompany the submission (17).

The revision-stage literature coverage audit, permanent-morbidity meta-analysis, population, era, and technique subgroup analyses, diagnostic-definition sensitivity analysis, and small-study analyses were added in response to peer review after registration. These additions are identified in the revised Methods and are interpreted as exploratory where appropriate; the registered review question and core eligibility criteria were unchanged.

### 2.2 Eligibility criteria

Eligible reports were original human studies describing stereotactic, stereotaxic, needle, frame-based, frameless, robotic, robot-assisted, or image-guided biopsy for brainstem lesions. No language restriction was applied. Conference abstracts were excluded from preferred quantitative pooling but could be retained as supplementary evidence when an extractable denominator was available. Because multiple publications could describe overlapping institutional cohorts, review-level eligibility was distinguished from inclusion in an outcome-specific quantitative synthesis.

### 2.3 Information sources and search strategy

The original search included PubMed/MEDLINE, ClinicalTrials.gov, Embase.com, Web of Science Core Collection, Scopus, Cochrane Library, CNKI, Wanfang, VIP, SinoMed, and Google Scholar through Publish or Perish, and was completed on 4 July 2026. During revision, the included studies in the Kickingereder 2013 and Hamisch 2017 meta-analyses were compared directly with the submitted evidence set, and forward and backward citation tracking was completed on 18 August 2026. Database-specific strategies and the revision-stage citation audit are provided in Supplementary Table S1 and the reproducibility materials.

### 2.4 Study selection

Records were deduplicated using DOI, normalized title, source metadata, and manual quality assurance. Title/abstract screening and full-text assessment followed a one-reviewer plus second-reviewer verification model, with disagreements resolved by discussion. Revision-stage candidates were eligible for quantitative inclusion only when the original report or an auditable primary-source extract supported the population, numerator, denominator, and outcome definition. Secondary-review event counts were not used as substitutes for primary data. Authors were not contacted to resolve two mixed or unclear population classifications.

### 2.5 Data extraction and outcome definitions

Data were extracted into outcome-specific workbooks covering study identity, population, enrollment period, biopsy technique, lesion location, diagnostic yield, overall complications, temporary and permanent neurological deficits, mortality, molecular testing, and clinical utility. Every quantitative row required an auditable numerator, denominator, denominator type, outcome definition, follow-up time point, and source location. Overall complications, temporary deficits, permanent deficits, and asymptomatic imaging findings were not treated as interchangeable outcomes.

Diagnostic yield was classified as histological or tissue adequacy, definitive or integrated diagnosis, or an author-reported diagnostic result. For Meshkini 2012, the preferred definition used 140 definitive diagnoses among 200 procedures; 60 presumptive diagnoses were retained only in a definition sensitivity analysis. For Chico-Ponce de Leon 2003, the text-consistent denominator of 50 was used, and the conflicting table label of 52 was documented without reconstructing unreported cases. For Gupta 2018, pathology-compatible diagnosis (48/50) and complete molecular assays (46/50) were recorded as separate constructs.

Permanent neurological morbidity required a deficit described by the source as permanent or still present at a clearly reported discharge or follow-up assessment. Studies with incompatible definitions or unresolvable follow-up were retained in a definition-stratified table or narrative synthesis rather than pooled. Dellaretti 2026 was not used for this outcome because its text and tables could not be reconciled.

### 2.6 Outcome synthesis framework

Diagnostic yield was synthesized separately from clinical utility because obtaining diagnostic tissue does not demonstrate treatment change or patient benefit. For overall safety, each study contributed one broad complication row selected by a prespecified hierarchy to limit within-study double counting. Permanent neurological morbidity and mortality were analyzed separately. Molecular testing success, complete molecular profiling, integrated diagnosis, target identification, treatment selection, and trial eligibility were retained as distinct constructs with their original denominators.

Publication from 2017 onward was used as a pragmatic era descriptor because 2017 was the first complete publication year after release of the WHO 2016 CNS classification and recognition of diffuse midline glioma, H3 K27M-mutant. This threshold was used to examine publication-era patterns and was not treated as a biological boundary. Explicit molecular testing was recorded independently of publication year.

### 2.7 Cohort overlap resolution

Potential overlap was resolved before meta-analysis using institution, authorship, enrollment period, population, technique, and sample size. The preferred representatives were Kratimenos 1993 for London, Puget 2015 for Necker, Peciu-Florianu 2022 for Lille, Chico-Ponce de Leon 2003 for Mexico, Rajshekhar 2010 for Vellore, Gupta 2018 for the North American DIPG cohort, and Meshkini 2012 for the Iranian brainstem series. Potentially overlapping reports were used only as replacements, definition sensitivity analyses, or narrative evidence and were never pooled simultaneously with their preferred lineage representative (Supplementary Table S2).

### 2.8 Risk of bias and certainty of evidence

Methodological quality was assessed with the JBI Case Series Checklist for uncontrolled surgical series and the JBI Cohort Studies Checklist for explicitly comparative cohorts. Judgments were recorded at item level as Yes, No, Unclear, or Not applicable, with no arbitrary numerical cutoff for excluding studies. Abstract-only records were not assigned a complete JBI appraisal. Item-level patterns informed outcome interpretation and GRADE certainty assessments (Supplementary Table S6).

### 2.9 Statistical analysis

Diagnostic yield and overall complications were synthesized with random-effects logit-transformed models using restricted maximum likelihood estimation. Study-level rows used observed proportions with exact binomial confidence intervals. Pooled estimates, 95% confidence intervals, prediction intervals, tau-squared, and I-squared were calculated, but I-squared was treated as descriptive and not as proof of clinical consistency.

Permanent neurological morbidity and procedure-related mortality were sparse and were analyzed primarily with binomial-normal GLMMs. Mortality was also reported as the crude aggregate proportion with an exact binomial 95% confidence interval. Continuity-corrected logit-REML, arcsine random-effects, fixed-effect logit, and Freeman-Tukey double-arcsine analyses were retained as sensitivity or method-boundary analyses; the Freeman-Tukey result was not used for inference.

Prespecified study-level subgroup analyses compared adult, pediatric, mixed, and mixed/unclear cohorts; publications before 2017 vs. 2017 onward; and robotic or robot-assisted studies vs. nonrobotic or not-reported-robotic studies. Technique categories were not mutually exclusive, so frame-based, frameless, and robotic series were not treated as three independent comparative groups. The robotic comparison was exploratory because it was confounded by era, center, and patient composition.

Sensitivity analyses excluded studies with fewer than 20 participants and stratified diagnostic yield by definition. The association between observed diagnostic yield and sample size was examined with Spearman correlation and exploratory study-level meta-regression; neither analysis was interpreted causally. Reporting-bias risk was assessed qualitatively from outcome availability and abstract-only evidence. Analyses were performed in R 4.5.1 using metafor 4.8-0; inputs, code, output, session information, and QA logs are included in the reproducibility materials.

## 3 Results

### 3.1 Study selection and revision-stage coverage audit

The original database/register search yielded 10,112 raw records and Google Scholar/Publish or Perish contributed 400 other-method records. After deduplication and manual quality assurance, 7,651 unique records formed the frozen title/abstract screening set. Seventy reports were assessed at full text; 14 were excluded, and 56 reports met review-level eligibility in the submitted review, including 51 full-text and 5 abstract/conference reports.

**Figure 1:**
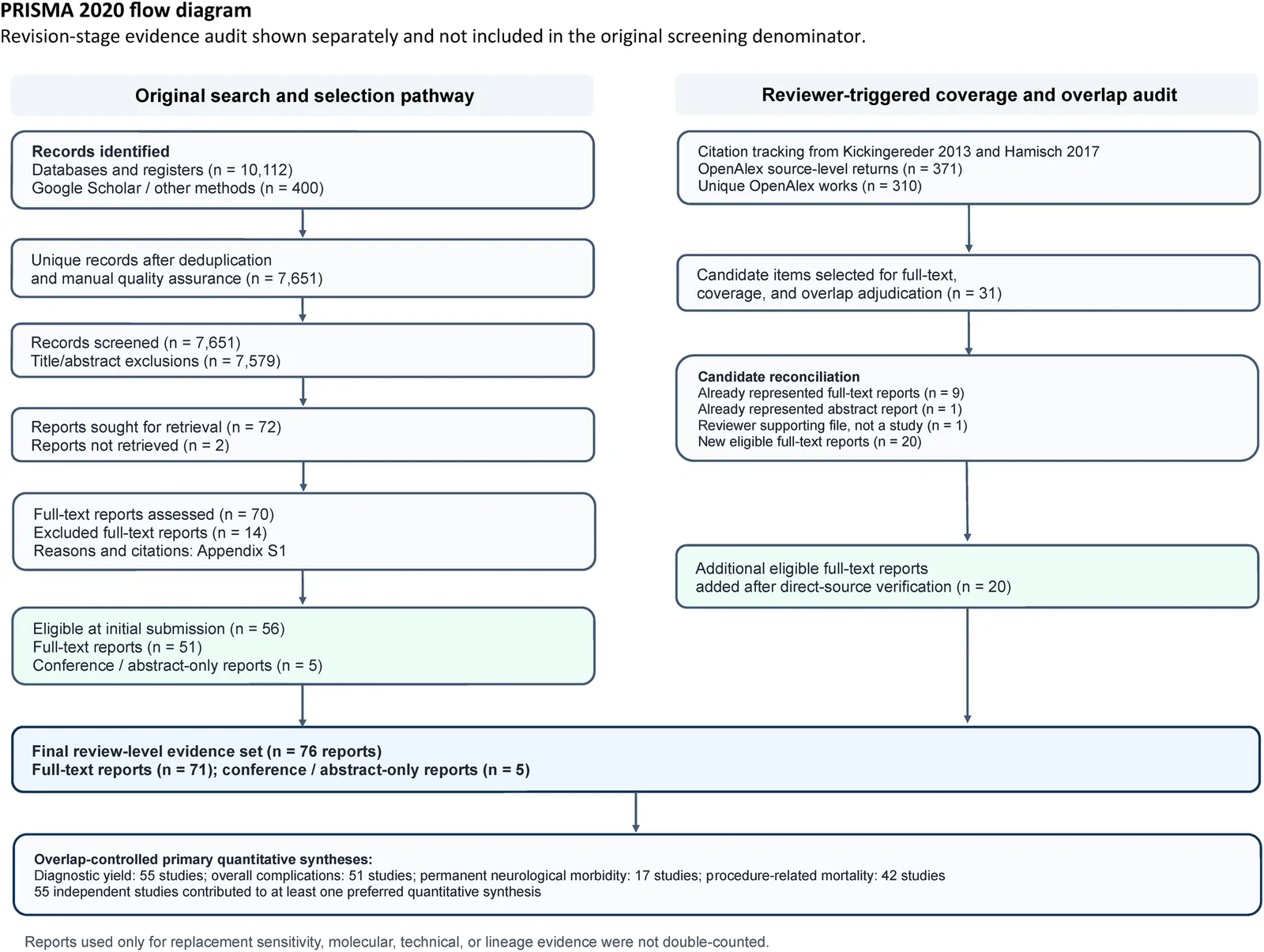
PRISMA 2020 flow diagram with revision-stage coverage audit. The diagram preserves the frozen original screening flow and separately reports the citation-tracking audit undertaken during revision. Twenty additional eligible full-text reports were added, yielding 76 review-level reports. Review-level reports are distinct from the 55 independent studies contributing to at least one preferred quantitative synthesis.

The submitted review contained 51 eligible full-text reports and 5 abstract/conference reports (18–62). The revision-stage comparison with the Kickingereder and Hamisch evidence sets and citation tracking identified 31 candidate items. Nine were already represented full-text reports, one was an already represented abstract, one was a nonstudy attachment, and 20 were additional eligible full-text reports (63–82). The final review-level evidence set therefore comprised 76 reports: 71 full-text reports and 5 abstract/conference reports. Fifty-five independent studies contributed to at least one preferred quantitative synthesis after outcome-specific overlap resolution. Figure 1 presents the original screening flow and the separate revision-stage coverage audit. Excluded full-text reports and reasons are listed in Appendix S1.

### 3.2 Study characteristics and overlap-controlled evidence

The 76 eligible reports were published from 1986 to 2026. Among the 55 primary quantitative studies, populations were adult in 9, pediatric in 18, mixed in 26, and mixed or unclear in 2. Frame-based methods were reported in 44, frameless methods in 15, and robotic or robot-assisted methods in 10 primary studies. Technique categories were not mutually exclusive because some reports included multiple approaches or historical transitions. The two mixed/unclear reports could not be classified from the publication, and authors were not contacted.

Outcome-specific selection retained the most informative nonduplicated report within each institutional lineage. Replacement analyses used Thomas 1988 for London, Dellaretti 2012 for Lille, Rajshekhar 1995 for Vellore, and Wang 2015 for the North American DIPG lineage without simultaneous inclusion of the preferred representative. The Lille primary analysis used Peciu-Florianu 2022, while Dellaretti 2026 represented a separate Brazilian center and enrollment period. Table 1 summarizes the evidence base, and Supplementary Table S2 provides report-level characteristics, outcome mapping, and overlap decisions.

**Table 1:** Study and evidence summary of included reports.

Domain	Summary value
Included evidence base	76 review-level eligible reports after revision-stage coverage and overlap audit: 71 full-text reports and 5 conference/abstract-only reports; publication years 1986–2026
Independent primary quantitative evidence	55 overlap-controlled studies contributed to at least one preferred quantitative synthesis; other eligible reports contributed replacement sensitivity, molecular, technical, or lineage evidence
Population categories among primary studies	Adult: 9; pediatric: 18; mixed: 26; mixed or unclear: 2
Diagnostic-yield analysis set	55 studies; 1,814/1,956 diagnostic outcomes; pooled estimate 93.5% (95% CI, 91.5%–95.0%)
Overall morbidity/complications analysis set	51 studies; 162/1,797 events; pooled estimate 10.5% (95% CI, 8.5%–12.8%)
Permanent neurological morbidity analysis set	17 studies; 14/859 events; GLMM estimate 0.8% (95% CI, 0.2%–3.0%)
Procedure-related mortality analysis set	42 studies; 11/1,765 deaths; crude proportion 0.6% (exact 95% CI, 0.3%–1.1%); primary GLMM estimate 0.3% (95% CI, 0.1%–1.2%)
Technique representation	Frame-based: 44; frameless: 15; robotic/robot-assisted: 10 primary studies. Categories are not mutually exclusive.
Molecular-era evidence	Publication from 2017 onward was treated as a pragmatic era descriptor; explicit molecular testing was recorded separately and synthesized narratively using construct-specific denominators

Review-level eligibility is distinct from inclusion in an outcome-specific quantitative synthesis. Population categories are study-level descriptors. Technique categories are not mutually exclusive. Overlap decisions and outcome-specific denominators are provided in Supplementary Table S2. CI, confidence interval; GLMM, generalized linear mixed model.

### 3.3 Diagnostic yield and small-study analyses

**Figure 2:**
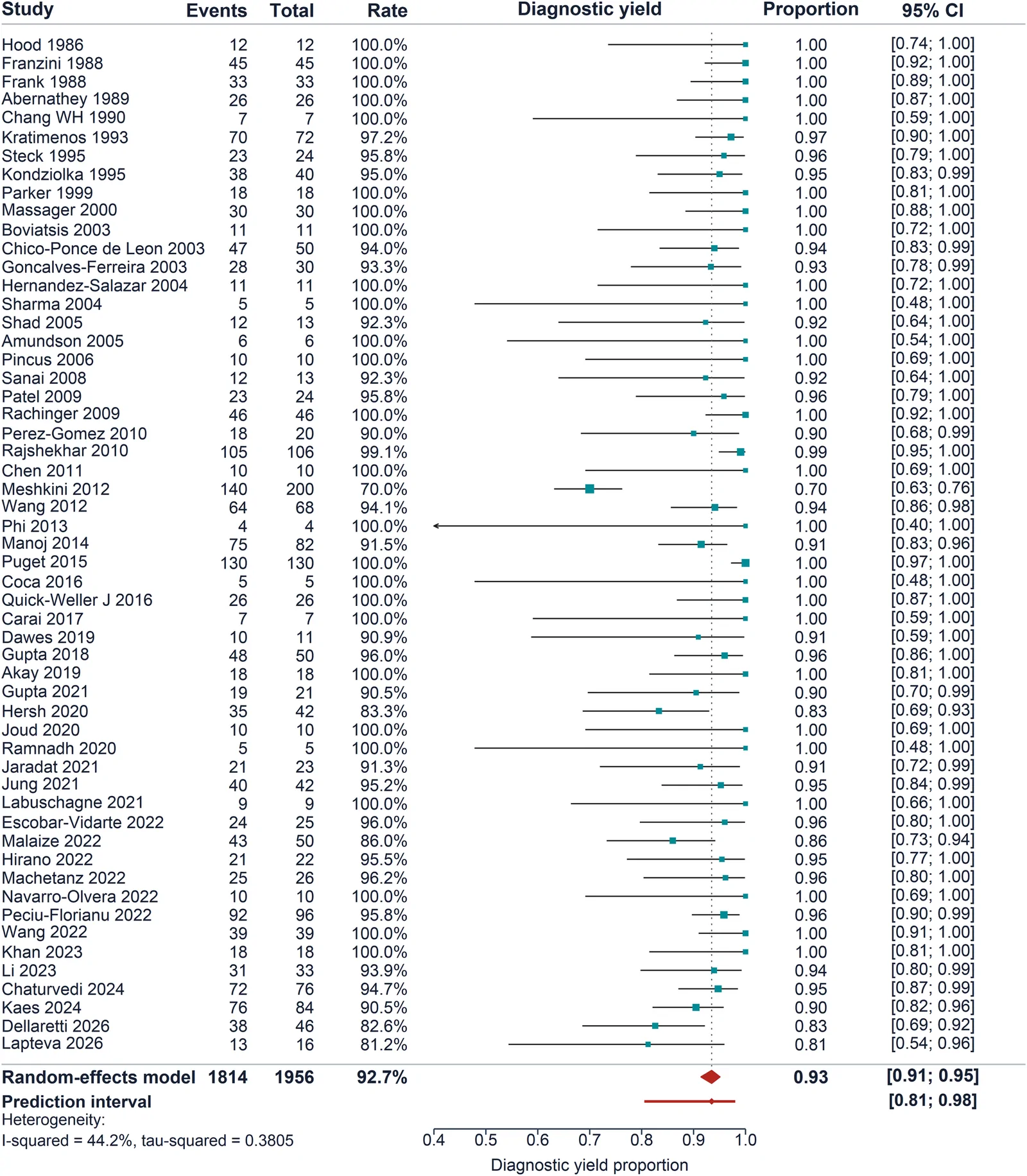
Forest plot of diagnostic yield after overlap resolution. Study-level rows show observed event/n proportions with exact binomial 95% confidence intervals. The pooled diamond, prediction interval, and heterogeneity statistics use the random-effects logit-REML model. Diagnostic yield was defined according to each report's extractable diagnostic-tissue or final-diagnosis denominator.

Diagnostic-yield data were available from 55 primary studies. Diagnostic tissue or a final diagnostic result was obtained in 1,814 of 1,956 patients or procedures (crude proportion 92.7%). The pooled diagnostic yield was 93.5% (95% CI, 91.5%–95.0%; 95% prediction interval, 80.5%–98.0%; *I*^2^ = 44.2%) (Figure 2; Table 2).

**Table 2:** Main pooled estimates.

Outcome	Studies	Events/denominator	Crude proportion	Primary model	Pooled proportion (95% CI)	95% prediction interval	*I* ^2^
Diagnostic yield	55	1,814/1,956	92.70%	logit random-effects REML	93.5% (91.5%–95.0%)	80.5%–98.0%	44.20%
Procedure-related morbidity/overall complications	51	162/1,797	9.00%	logit random-effects REML	10.5% (8.5%–12.8%)	4.3%–23.1%	37.40%
Permanent neurological morbidity	17	14/859	1.63%	binomial-normal GLMM	0.83% (0.22%–3.03%)	0.06%–10.84%	59.30%
Procedure-related mortality	42	11/1,765	0.62%	binomial-normal GLMM	0.30% (0.07%–1.24%)	0.02%–5.31%	52.20%

Diagnostic yield and overall complications used logit-transformed REML random-effects models. Permanent morbidity and mortality used binomial-normal GLMMs because events were sparse. The crude proportion is the aggregate event count divided by the aggregate denominator. For mortality, the crude exact-binomial estimate and GLMM are primary; continuity-corrected logit, arcsine, and Freeman-Tukey boundary analyses are reported in Supplementary Table S4. *I*^2^ is descriptive and is not interpreted as proof of consistency for sparse-event outcomes. CI, confidence interval; GLMM, generalized linear mixed model; REML, restricted maximum likelihood.

Population subgroup estimates were 91.6% (95% CI, 86.0%–95.1%) in 9 adult studies, 94.1% (90.5%–96.4%) in 18 pediatric studies, 94.2% (92.0%–95.8%) in 26 mixed studies, and 70.7% (64.3%–76.4%) in 2 mixed/unclear studies. Estimates before 2017 and from 2017 onward were 94.8% (92.1%–96.6%) and 91.8% (88.9%–94.0%), respectively. Robotic or robot-assisted studies had an exploratory estimate of 94.8% (91.1%–97.0%), compared with 93.3% (91.0%–95.1%) for nonrobotic or not-reported-robotic studies (Supplementary Table S5).

When studies with fewer than 20 participants were excluded, the diagnostic-yield estimate remained 93.7% (95% CI, 91.3%–95.5%; 33 studies, 1,591/1,727). Definition-stratified estimates were 93.6% for histological or tissue adequacy, 91.9% for definitive or integrated diagnosis, and 93.7% for author-reported diagnostic results. Observed study-level yield decreased with sample size (Spearman rho = −0.47, *p* = 0.0003); exploratory meta-regression showed the same direction (logit slope per participant −0.0066, *p* = 0.0185). Supplementary Figure S1 displays this relationship. These findings support a small-study or selective-reporting concern but do not quantify publication bias causally.

### 3.4 Overall complications, permanent morbidity, and mortality

**Figure 3:**
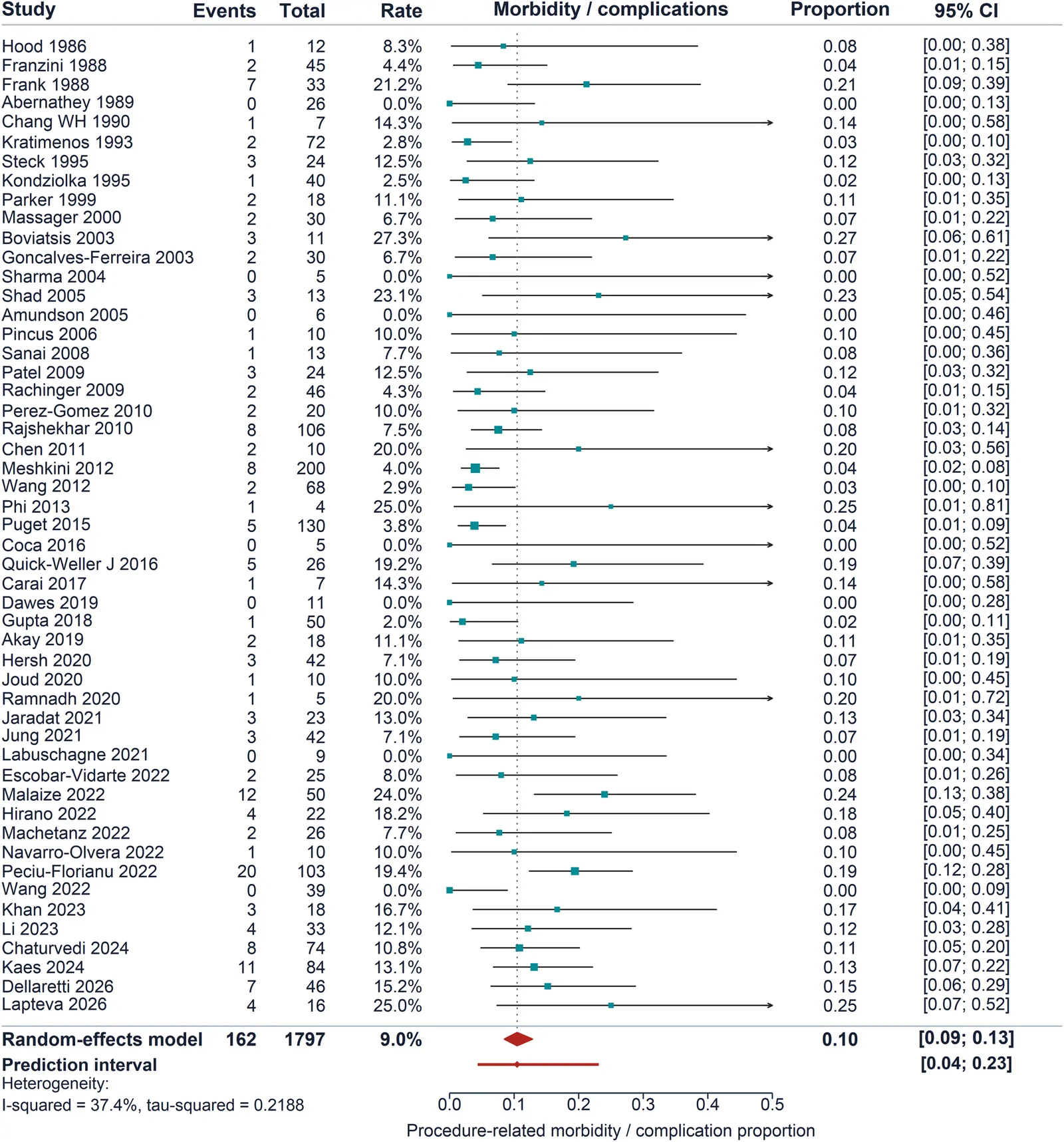
Forest plot of procedure-related morbidity or overall complications. Each study contributed one broad safety row selected by the prespecified hierarchy. Study-level rows show exact binomial intervals; the pooled estimate uses the random-effects logit-REML model. Event composition and follow-up varied, so the estimate should be interpreted with the separate permanent-morbidity analysis.

Overall procedure-related morbidity or complications were available from 51 primary studies. Across 1,797 patients or procedures, 162 events were reported (crude proportion 9.0%). The pooled estimate was 10.5% (95% CI, 8.5%–12.8%; 95% prediction interval, 4.3%–23.1%; *I*^2^ = 37.4%) (Figure 3; Table 2). The corresponding estimates were 13.8% in adult, 7.7% in pediatric, 11.5% in mixed, and 10.1% in mixed/unclear studies. Estimates were 8.7% before 2017 and 13.5% from 2017 onward. Robotic or robot-assisted studies had an exploratory estimate of 13.4%, compared with 10.1% in other studies. Excluding studies with fewer than 20 participants yielded 9.1% (95% CI, 7.0%–11.8%) (Supplementary Table S5).

Seventeen studies provided compatible permanent-neurological-morbidity data. Fourteen events occurred among 859 patients or procedures (crude 1.63%, exact 95% CI, 0.89%–2.72%). The primary GLMM estimate was 0.83% (95% CI, 0.22%–3.03%; 95% prediction interval, 0.06%–10.84%). Definitions ranged from author-defined permanent deficits to deficits still present at discharge or follow-up, so the prediction interval and certainty rating were emphasized rather than treating 0.83% as a universal counseling estimate (Table 2; Supplementary Table S2).

**Figure 4:**
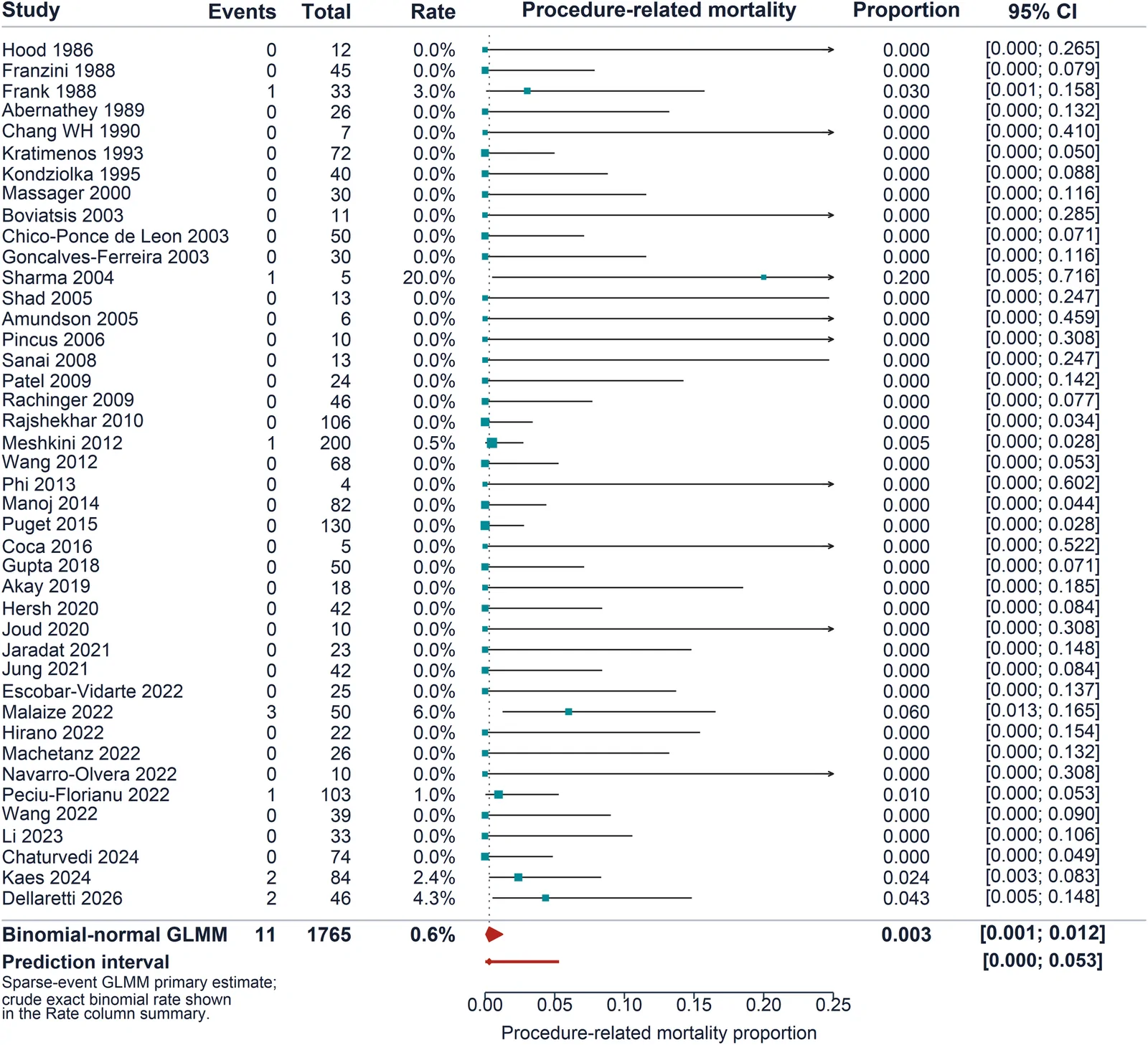
Forest plot of procedure-related mortality using the primary binomial-normal GLMM. Study-level rows show observed event/n proportions with exact binomial 95% confidence intervals. The pooled diamond and prediction interval use the binomial-normal GLMM. The crude aggregate proportion was 11/1,765 (0.62%, exact 95% CI, 0.31%–1.11%). Continuity-corrected logit, arcsine, and Freeman-Tukey boundary analyses are reported in Supplementary Table S4. *I*^2^ is descriptive and is not evidence of consistency for sparse events.

Procedure-related mortality was reported or reliably inferable in 42 studies. Eleven deaths occurred among 1,765 patients or procedures, giving a crude proportion of 0.62% (exact 95% CI, 0.31%–1.11%). The primary binomial-normal GLMM estimate was 0.30% (95% CI, 0.07%–1.24%; 95% prediction interval, 0.02%–5.31%) (Figure 4). The continuity-corrected logit-REML sensitivity estimate was 2.31%, whereas the arcsine estimate was 0.14%; the Freeman-Tukey boundary test collapsed toward zero and was not used for inference (Supplementary Table S4). The observed mortality proportion was therefore below 1%, while model-based uncertainty remained substantial. *I*^2^ was not interpreted as evidence of event consistency.

### 3.5 Molecular-era clinical utility

Molecular-era evidence remained heterogeneous. Fourteen reports explicitly reported molecular testing, including histone alteration testing, methylation or sequencing-based profiling, targetable alteration identification, integrated diagnosis refinement, treatment selection, and trial eligibility. Denominators differed across all-biopsy cohorts, tumor-only subsets, samples submitted for testing, successfully profiled samples, and mixed-technique cohorts; these endpoints were not combined into one pooled utility estimate (Supplementary Table S3).

The underlying numbers represented different stages of the tissue-to-decision pathway. Carai et al. reported molecular targets available in 7/7 patients (9), Khan et al. tested all 18 biopsy samples (16), the INFORM cohort completed comprehensive profiling in 21/21 enrolled biopsy patients (45), and Wang et al. reported molecular analysis in 39/39 robot-assisted cases (53). In the prospective North American DIPG cohort, pathology was compatible with the radiographic diagnosis in 48/50 patients and complete molecular assays were obtained in 46/50; these were distinct outcomes (75). By contrast, 13/88 in Fagundes et al. and 10/96 in Peciu-Florianu et al. denoted tested subsets of longer cohorts rather than testing success in all patients (14, 38).

### 3.6 Risk of bias and certainty of evidence

All 55 primary quantitative studies underwent item-level JBI appraisal: 51 uncontrolled series used the Case Series Checklist and 4 explicitly comparative cohorts used the Cohort Checklist. Reporting of condition measurement, diagnostic methods, clinical information, and outcomes was generally adequate. Consecutive inclusion was unclear in 31 of 51 uncontrolled series, complete inclusion was unclear in 8, and several reports incompletely described demographics, site characteristics, or statistical methods. Among comparative cohorts, confounding control or follow-up handling was incompletely reported in one study. Detailed judgments and source locations are provided in Supplementary Table S6.

GRADE certainty was low for diagnostic yield and overall complications, and very low for permanent neurological morbidity, mortality, and molecular-era clinical utility. Downgrading reflected observational design, selection and reporting concerns, outcome-definition variation, sparse events, imprecision, and heterogeneous molecular constructs (Table 3).

**Table 3:** GRADE certainty summary and interpretation boundaries.

Outcome	Studies/participants or procedures	Certainty	Main reasons for downgrading
Diagnostic yield	55 studies/1,956 patients or procedures	Low	Observational evidence; selection and reporting concerns; varying diagnostic-yield definitions.
Procedure-related morbidity/overall complications	51 studies/1,797 patients or procedures	Low	Observational evidence; variable event composition, ascertainment, and follow-up.
Permanent neurological morbidity	17 studies/859 patients or procedures	Very low	Observational evidence; sparse events; serious inconsistency in definitions and follow-up time points.
Procedure-related mortality	42 studies/1,765 patients or procedures	Very low	Observational evidence; very sparse events; model sensitivity and incomplete outcome reporting.
Molecular-era clinical utility	14 reports with explicit molecular testing/heterogeneous denominators	Very low	Observational evidence; indirectness; heterogeneous testing constructs, denominators, and downstream endpoints.

GRADE, Grading of Recommendations Assessment, Development and Evaluation. Certainty judgments apply to the body of observational evidence and should not be read as a recommendation for routine biopsy. Permanent-morbidity definitions ranged from author-defined permanent deficits to deficits still present at discharge or follow-up. Molecular-era clinical utility was synthesized narratively because testing and management denominators were not exchangeable.

### 3.7 Reporting bias assessment

Outcome availability differed across eligible reports, and five abstract-only records lacked full methods. The inverse association between observed diagnostic yield and study size and the concentration of 100% yields among small series supported concern about selective publication or reporting. However, the diagnostic estimate changed little after excluding studies with fewer than 20 participants. Conventional funnel-plot asymmetry tests were not interpreted because heterogeneous single-arm proportions and sparse events violate their usual assumptions.

## 4 Discussion

### 4.1 Principal findings

After rebuilding the coverage and overlap audit, this review included 76 eligible reports and 55 independent studies in at least one preferred quantitative synthesis. Stereotactic biopsy showed a pooled diagnostic yield of 93.5% and an overall complication estimate of 10.5%. Permanent neurological morbidity was estimated at 0.83%, although the confidence and prediction intervals were wide. Procedure-related mortality was observed in 0.62% of patients or procedures and estimated at 0.30% by the primary GLMM. These data support biopsy as an option for selected patients in experienced settings, not as a routine intervention for every brainstem lesion.

### 4.2 Diagnostic yield, sample adequacy, and small-study effects

Imaging and tissue answer different clinical questions. Imaging defines anatomy, pattern, and procedural feasibility, but it may not reliably distinguish glioma grade from lymphoma, metastasis, inflammation, infection, or other mimics in every patient. Tissue is most defensible when a plausible diagnosis would redirect treatment, prognosis, or trial eligibility (4–6).

A high pooled yield is not a guarantee for an individual procedure. Failure can arise from sampling necrotic or cystic components, avoiding vascular or eloquent regions at the expense of the most informative target, intralesional heterogeneity, trajectory or navigation error, small or mechanically damaged cores, and delays or mismatch in specimen handling. These mechanisms were not reported consistently enough for comparative pooling, but they are clinically relevant when planning the target and tissue workflow.

Intraoperative smear or frozen-section assessment can confirm that lesional tissue has been obtained and permit additional sampling while the trajectory remains available. Intraoperative imaging may verify targeting, and fluorescence-based *ex vivo* inspection has been explored in contrast-enhancing lesions. Its relevance to nonenhancing diffuse pontine or midline glioma remains uncertain, and the present brainstem-specific evidence does not support routine fluorescence use. These adjuncts should be presented as context-dependent quality-control options rather than proven determinants of superior outcome (78, 83).

The inverse study-size association suggests that small favorable series contribute selective-reporting risk. The estimate remained similar after excluding studies with fewer than 20 participants, which argues against complete dependence on the smallest reports, but does not remove publication bias. The lower crude yield than the transformed pooled estimate and the broad prediction interval should remain visible in interpretation.

### 4.3 Population, era, and technique

Adult, pediatric, and mixed cohorts produced broadly high pooled yields, but they address different diagnostic pathways. Pediatric evidence is dominated by diffuse pontine or midline tumors and molecular trial questions, whereas adult lesions have a wider pathological differential. Population subgroup estimates are ecological study-level summaries and should not be interpreted as direct age effects.

The 2017 era comparison did not show a higher pooled diagnostic yield in later publications, while overall complication reporting was numerically higher after 2017. This should not be read as deterioration in modern safety. Later studies may use more intensive imaging surveillance, broader adverse-event definitions, and more challenging molecularly selected populations. The threshold marks the first complete publication year after WHO 2016 and is a practical reporting boundary rather than a biological transition.

Robotic series had high diagnostic yield but did not show an unambiguously lower complication estimate. Robotic and nonrobotic categories were confounded by era, center experience, lesion type, and patient selection. Frame-based, frameless, and robotic methods also overlapped within reports, so a three-way comparison would create false independence. Platform and trajectory should be chosen according to target anatomy, verified local accuracy, team experience, and complication-response capacity rather than an assumed universal ranking.

### 4.4 Interpreting safety for clinical counseling

The broad overall-complication outcome intentionally captures the burden reported by each study, but it combines events of different severity and duration. An asymptomatic hemorrhage detected on routine imaging is not equivalent to a persistent bulbar deficit. The separate permanent-morbidity analysis responds to this clinical distinction and provides a more relevant counseling endpoint, while its wide prediction interval reflects inconsistent definitions and follow-up.

For counseling, the overall complication estimate should therefore be accompanied by the permanent-deficit and mortality results and by local outcome data. Published cohorts were selected and frequently came from experienced centers. A transparent discussion should distinguish transient neurological worsening, persistent neurological injury, hemorrhage requiring intervention, and death, and should state the follow-up interval used by the local service.

The revised mortality analysis removes the continuity-corrected logit estimate from the primary position. With 11 deaths among 1,765 patients or procedures, the observed proportion was 0.62%. The GLMM estimate was 0.30%, whereas the continuity-corrected logit model was 2.31%. The latter weights sparse zero-event studies after correction and is best interpreted as a sensitivity boundary rather than the clinical event rate. Conversely, the Freeman-Tukey result shrank toward zero and was also unsuitable for primary inference. The crude exact interval and GLMM together provide the clearest account of a rare but nonzero event.

### 4.5 Molecular-era clinical utility

The contemporary rationale for biopsy extends beyond morphology. Integrated CNS tumor classification, molecular profiling, and biomarker-stratified trials can make tissue clinically consequential even when imaging strongly suggests a diagnosis (5, 7, 8, 45). However, molecular testing success, complete profiling, diagnostic refinement, identification of a target, treatment change, and survival benefit are different outcomes.

This distinction explains why the molecular evidence was not pooled. A successful H3 K27 assay in a selected sample does not equal a complete methylation or sequencing profile, and neither necessarily changes treatment. Clinical utility also depends on preanalytic handling, neuropathology review, assay turnaround, access to targeted agents, and trial availability. The evidence supports feasibility and structured decision support in selected settings, but not a single universal utility rate or a claim of improved survival.

### 4.6 Clinical decision framework

A practical multidisciplinary decision should begin with the tissue-dependent question. The team should document the expected information gain, the safest feasible trajectory, the probability that each plausible result would alter management, and whether pathology and molecular workflows can use the specimen. Neurological status, lesion location, imaging pattern, anesthetic risk, center experience, and available therapeutic pathways should be considered together. If tissue is unlikely to change diagnosis, treatment, prognosis, or trial access, observation or noninvasive reassessment may be preferable.

### 4.7 Relation to previous evidence

The submitted review initially contained fewer pooled patients than the Kickingereder 2013 and Hamisch 2017 meta-analyses. Direct comparison identified omitted original reports, different brainstem-specific denominators, conference records, and institutional overlap choices. The revision added 20 verified full-text reports while retaining one representative per potentially overlapping lineage (63–82). This increased the diagnostic analysis to 1,956 patients or procedures and made the coverage difference explicit rather than importing secondary-review totals.

The revised analysis extends earlier evidence by adding a separately pooled permanent-morbidity outcome, placing the mortality GLMM and crude exact estimate in the primary position, examining population and era subgroups, testing diagnostic definitions and small-study sensitivity, and mapping molecular outcomes by construct. These additions improve comparability with prior reviews while preserving caution about observational evidence.

### 4.8 Strengths and limitations

Strengths include a broad multilingual search, direct comparison with prior meta-analyses, forward and backward citation tracking, auditable outcome-specific extraction, explicit cohort-lineage control, multiple sparse-event models, permanent-morbidity pooling, subgroup and definition sensitivity analyses, and reproducible code and inputs. Distinguishing 76 review-level reports from 55 independent primary studies prevents report counts from being confused with pooled datasets.

Limitations remain substantial. Most evidence came from retrospective or uncontrolled series of selected patients. Consecutive and complete inclusion were often incompletely reported. Diagnostic, complication, and permanent-morbidity definitions and follow-up intervals varied. Patient and procedure denominators were not always interchangeable. The population, era, and robotic analyses were ecological and confounded. Small favorable reports may be preferentially published. Seven non-primary overlapping or narrative reports did not have a retained source file for complete revision-stage JBI reappraisal, and five abstract-only records lacked full methods; neither group drove the preferred quantitative estimates. Remaining uncertainty about North American site-level overlap was handled conservatively by not pooling Wang 2015 with Gupta 2018.

Molecular-era endpoints were particularly diverse, and most reports did not trace the complete pathway from tissue acquisition through testing, management change, and patient outcome. The findings therefore describe performance in selected published cohorts rather than comparative superiority over nonbiopsy management or one optimal technical platform.

### 4.9 Research implications

Future prospective registries should use explicit patient and procedure denominators and distinguish tissue adequacy, histological diagnosis, integrated molecular diagnosis, transient deficit, persistent deficit at standardized follow-up, asymptomatic hemorrhage, major intervention-requiring morbidity, and death. Reports should also record target and trajectory, intraoperative sample verification, platform accuracy, pathology workflow, molecular assay completion, turnaround time, treatment change, and trial enrollment. Such reporting would support clinically meaningful comparisons without treating all lesions, techniques, or molecular endpoints as interchangeable.

## 5 Conclusion

In selected published cohorts, stereotactic biopsy for brainstem lesions provided high diagnostic yield. Overall complications were clinically meaningful, whereas permanent neurological morbidity was uncommon but imprecisely estimated. Procedure-related mortality was observed in fewer than 1% of patients or procedures; the primary GLMM estimate was lower than the continuity-corrected logit sensitivity estimate, underscoring rare-event model uncertainty. Population, era, technique, diagnostic-definition, and small-study analyses support individualized multidisciplinary decision-making rather than routine biopsy for all brainstem lesions. Molecular evidence supports tissue-enabled diagnosis and decision support in selected settings, but not a single pooled utility rate or an assumption of improved survival.

## Data Availability

The original contributions presented in the study are included in the article/Supplementary Material, further inquiries can be directed to the corresponding author/s.

## Supplementary material

Supplementary Figure S1 Points are colored by the prespecified n = 20 sensitivity threshold. The negative Spearman correlation is descriptive and may reflect selective publication, study composition, or other ecological differences; it is not a patient-level causal effect.
