# Supplementary material for: Diagnostic yield, safety, and molecular-era clinical utility of stereotactic biopsy for brainstem lesions: a contemporary systematic review and meta-analysis
Source: Front Surg. 2026 Sep 9;13:1953479. doi: 10.3389/fsurg.2026.1953479 (PMC13597709; doi:10.3389/fsurg.2026.1953479)
Supplement: Supplementary file 1 [file Table7.docx]

**Appendix S1. Excluded full-text reports and reasons**

Fourteen full-text records were excluded during the frozen original eligibility assessment. Original-language Chinese titles are retained with bracketed English explanatory translations, and record identifiers distinguish duplicate screened records. Exclusion counts were duplicate or overlapping reports, n = 6; no original clinical data, n = 5; and insufficient extractable data, n = 3. The separate revision-stage coverage audit identified 20 additional eligible full-text reports; it did not add another excluded full-text report. The Reviewer 2 supporting attachment was administrative peer-review material rather than a candidate study.

| **No.** | **Report citation and screened record ID** | **Reason for exclusion** |
| --- | --- | --- |
| 1 | Beynon C, Kiening KL. Stereotactic biopsy of brainstem lesions: a 'golden standard' for establishing the diagnosis. J Neurosci Rural Pract. 2014;5(1):9-10. doi: 10.4103/0976-3147.127863.  *Screened record ID: b86da3cee3e70659* | No original clinical data; editorial/commentary. |
| 2 | Rajshekhar V. Stereotactic biopsy of brain stem masses: a safe and useful procedure. J Neurosci Rural Pract. 2014;5(1):8. doi: 10.4103/0976-3147.127862.  *Screened record ID: 6253c87e1e66784f* | No original clinical data; editorial/commentary. |
| 3 | Sheikh SR, Recinos VMR, Thompson EM, et al. The role of brainstem biopsy and targeted therapies in pediatric diffuse midline glioma/diffuse intrinsic pontine glioma. Front Oncol. 2024;14:1504440. doi: 10.3389/fonc.2024.1504440.  *Screened record ID: 11e2f44a72f70354* | No original clinical data; narrative review. |
| 4 | Marupudi NI, Rostomian L, McVeigh L, Sood S. Intraoperative electromyography of cranial nerves to reduce risk of neurologic injury in stereotactic biopsy of diffuse intrinsic pontine glioma. Meeting-abstract record associated with the 48th Annual Meeting of the American Society of Pediatric Neurosurgeons. J Neurosurg Pediatr. 2025.  *Screened record ID: 04c3d27e6ae94c06* | Insufficient extractable data; the retrieved meeting preview did not contain the target abstract or full report. |
| 5 | Ning QF, translator. 脑干局灶性病变的立体定向活检 [Stereotactic biopsy of focal brainstem lesions]. 中国微侵袭神经外科杂志 [Chinese Journal of Minimally Invasive Neurosurgery; translated journal title]. 2004. [in Chinese]  *Screened record ID: 78dffc3205d8152e* | Duplicate/translation-like report of an original cohort retained elsewhere. |
| 6 | Wang W. MRI结合PET引导下脑干占位性病变立体定向活检30例报告 [MRI- and PET-guided stereotactic biopsy of brainstem mass lesions: report of 30 cases]. 中国微侵袭神经外科杂志 [Chinese Journal of Minimally Invasive Neurosurgery; translated journal title]. 2001. [in Chinese]  *Screened record ID: 904e6181cafdedf8* | Duplicate/translation-like report of an original cohort retained elsewhere. |
| 7 | Chang QY. 脑干占位病变的立体定向活检 [Stereotactic biopsy of brainstem mass lesions]. 国外医学·神经病学神经外科学分册 [Foreign Medical Sciences: Section of Neurology and Neurosurgery; translated journal title]. 1997. [in Chinese]  *Screened record ID: b65a73de209ff93f* | Duplicate/translation-like report of an original cohort retained elsewhere. |
| 8 | Chen XG. 脑干内部病变的立体定向活检 [Stereotactic biopsy of intrinsic brainstem lesions]. 国外医学·神经病学神经外科学分册 [Foreign Medical Sciences: Section of Neurology and Neurosurgery; translated journal title]. 1988. [in Chinese]  *Screened record ID: 3761121b0dbec6e4* | Duplicate/translation-like report of an original article retained elsewhere. |
| 9 | Patt S, Weigel K, Mayer HM. A case of dyschondroplasia associated with brain stem glioma: diagnosis by stereotactic biopsy. Neurosurgery. 1990;27(3):487-491. doi: 10.1227/00006123-199009000-00029.  *Screened record ID: 8e41a98ee4e859e8* | Insufficient extractable data; single-case report. |
| 10 | Chang QY. 脑干占位病变的立体定向活检 [Stereotactic biopsy of brainstem mass lesions]. 国外医学·神经病学神经外科学分册 [Foreign Medical Sciences: Section of Neurology and Neurosurgery; translated journal title]. 1997. [in Chinese]  *Screened record ID: 29354744449636d9* | Duplicate screened record of the same translation-like report (record b65a73de209ff93f). |
| 11 | Dawes W, Marcus HJ, Tisdall M, Aquilina K. Robot-assisted stereotactic brainstem biopsy in children: prospective cohort study. J Robot Surg. 2019;13(4):575-579. doi: 10.1007/s11701-018-0899-x.  *Screened record ID: 3e25cd9d7d73c7b9* | Duplicate bibliographic/full-text representation of the retained Dawes cohort. |
| 12 | Mathon B, Malaizé H, Amelot A, PSL BRAIN-BIOPSY Study Group. Stereotactic biopsies of brainstem lesions: dilemma on the best trajectory. Acta Neurochir (Wien). 2022;164(3):745-746. doi: 10.1007/s00701-021-04924-x.  *Screened record ID: 0bea873eaec3290c* | No original clinical data; letter commenting on another cohort. |
| 13 | Schlaeppi JA, Andereggen L, Nowacki A, Pollo C. Stereotactic posterior midline approach under direct microscopic view for biopsy of medulla oblongata tumors: technical considerations. Acta Neurochir (Wien). 2021;163(7):1965-1968. doi: 10.1007/s00701-020-04600-6.  *Screened record ID: 6b40c6c7d19936c2* | Insufficient extractable data; single-case technical report. |
| 14 | Yang CC, Wei XP. 立体定向活检术在脑干病变中的应用价值 [Clinical value of stereotactic biopsy for brainstem lesions]. 立体定向和功能性神经外科杂志 [Chinese Journal of Stereotactic and Functional Neurosurgery; translated journal title]. 2011;24(4):253-256. [in Chinese]  *Screened record ID: 2db907f3be3f5d42* | No original clinical data; Chinese-language narrative review. |
